# Autophagic-Lysosomal Inhibition Compromises Ubiquitin-Proteasome System Performance in a p62 Dependent Manner in Cardiomyocytes

**DOI:** 10.1371/journal.pone.0100715

**Published:** 2014-06-24

**Authors:** Zongwen Tian, Changhua Wang, Chengjun Hu, Yihao Tian, Jinbao Liu, Xuejun Wang

**Affiliations:** 1 Division of Basic Biomedical Sciences, Sanford School of Medicine of the University of South Dakota, Vermillion, South Dakota, United States of America; 2 Protein Modification and Degradation Laboratory, Departments of Pathophysiology and Biochemistry, Guangzhou Medical University, Guangzhou, Guangdong, China; Mayo Clinic, United States of America

## Abstract

Intracellular protein degradation is primarily performed by the ubiquitin-proteasome system (UPS) and the autophagic-lysosomal pathway (ALP). The interplay between these two pathways has been rarely examined in intact animals and the mechanism underlying the interplay remains unclear. Hence, we sought to test *in vivo* and *in vitro* the impact of inhibition of the ALP on UPS proteolytic performance in cardiomyocytes and to explore the underlying mechanism. Transgenic mice ubiquitously expressing a surrogate UPS substrate (GFPdgn) were treated with bafilomycin-A1 (BFA) to inhibit the ALP. Myocardial and renal GFPdgn protein levels but not mRNA levels were increased at 24 hours but not 3 hours after the first injection of BFA. Myocardial protein abundance of key proteasome subunits and the activities of proteasomal peptidases were not discernibly altered by the treatment. In cultured neonatal rat ventricular myocytes (NRVMs), the surrogate UPS substrate GFPu and a control red fluorescence protein (RFP) were co-expressed to probe UPS performance. At 12 hours or 24 hours after ALP inhibition by 3-methyladenine (3-MA) or BFA, GFPu/RFP protein ratios and the protein half-life of GFPu were significantly increased, which is accompanied by increases in p62 proteins. Similar findings were obtained when ALP was inhibited genetically via silencing Atg7 or Rab7. ALP inhibition-induced increases in GFPu and p62 are co-localized in NRVMs. siRNA-mediated p62 knockdown prevented ALP inhibition from inducing GFPu accumulation in NRVMs. We conclude that in a p62-dependent fashion, ALP inhibition impairs cardiac UPS proteolytic performance in cardiomyocytes *in vitro* and *in vivo*.

## Introduction

The degradation of most proteins, native or misfolded, in the cell is carried out by the ubiquitin-proteasome system (UPS). UPS-mediated protein degradation involves two main steps: ubiquitination of individual substrate protein molecules and degradation of the ubiquitinated proteins by the proteasome [1]. UPS dysfunction, especially proteasome functional insufficiency (PFI) [2]–[4], has been implicated in cardiac pathogenesis [5], [6]; hence, identification of the factors that impact on proteasome functioning in cardiomyocytes and in the heart is expected to improve our understanding of the molecular mechanisms underlying the progression from various types of heart disease to congestive heart failure, the final common pathway of virtually all cardiovascular disease and the leading cause of death in humans.

The autophagic-lysosomal pathway (ALP) represents another major cytoplasmic catabolic pathway that delivers cytoplasmic components (including organelles) to the lysosome for degradation [1]. Based on the route via which a substrate is delivered to the lysosomal lumen, autophagy is divided into three categories: microautophagy, chaperone-mediated autophagy (CMA), and macroautophagy. In microautophagy, the lysosome uptakes substrates by the invagination of the lysosomal membrane to engulf cytoplasmic material directly into the lysosome lumen. The CMA is a specific pathway in which the individual target protein molecules bearing the CMA recognition sequence (a KFERQ motif or KFERQ-like motif) is recognized and bound by the heat shock cognate protein 70 (Hsc70) complex, and is subsequently transferred to the lysosomal membrane where the substrate protein is translocated into lysosomal lumen via a complex formed primarily by lysosomal membrane associated protein 2A (LAMP-2A) [7]. Differing from microautophagy and CMA, macroautophagy requires formation of an autophagosome to target bulky cytoplasmic materials including organelles or protein aggregates to lysosomes for degradation. By self-digestion of a portion of cytoplasm to provide energy and essential amino acids for the synthesis of important proteins, macroautophagy helps cell to survive during nutrient deprivation. Macroautophagy can also selectively degrade damaged or surplus organelles as well as aberrant protein aggregates; hence, macroautophagy plays an important role in both organelle quality control and protein quality control in the cell. Macroautophagy is the most studied and the best understood type of autophagy; hence, it is commonly referred to as autophagy. It becomes increasingly clear that changes in autophagy are involved in the regulation of a variety of physiological and pathophysiological processes in the heart [8]–[14]. Emerging data suggest that inadequate ALP function may contribute to cardiac pathogenesis, especially the development of congestive heart failure [3], [15].

The proteasomal and lysosomal degradation were historically regarded as two parallel pathways but emerging evidence suggests that the two pathways actually interact with each other and their interplay may be critical to maintaining proteostasis in the cell [16], [17]. Proteasome inhibition has been shown to activate macroautophagy in cultured cells and intact mice [18]. However, very few studies have examined the *in vivo* effect of inhibition of the ALP on UPS function. The few reported studies, which used only cell cultures, came to contradictory conclusions. Korolchuk *et al*. showed in cultured HeLa cells and mouse embryonic fibroblasts (MEF) that pharmacological or genetic inhibition of autophagy does not alter proteasome peptidase activities but hinders the degradation of ubiquitinated substrates by the proteasome due to accumulation of p62/SQUSTM1 [19]. More recently, Wang *et al*. reported that inhibition of autophagy leads to increased proteasome activities and upregulation of proteasomal subunits in cultured human colon cancer cells under nutrient-deficient conditions [20]. Notably, these reported findings have not been tested in intact animals and also importantly, it remains unclear whether and how impaired ALP impacts on UPS-mediated proteolysis in cardiomyocytes.

In the present study, we have examined the impact of inhibition of the ALP at the autophagosome formation or removal stages on UPS proteolytic function using a mouse model of an inverse reporter of UPS function. We also investigated the effect of the inhibition of the ALP on UPS function in cultured cardiomyocytes. Our results indicate that inhibition of the ALP causes a delayed impairment of UPS performance in a p62-dependent manner in cardiomyocytes.

## Methods

### 1. Animals and treatments

This investigation conforms with the Guide for the Care and Use of Laboratory Animals published by the US National Institutes of Health (NIH Publication No. 85-23, revised 1996). It was also approved by the Institutional Animal Care and Use Committee of the University of South Dakota, Vermillion, SD, USA. The creation and initial characterization of transgenic (tg) mice which constitutively and ubiquitously express an enhanced green fluorescence protein (GFP) that is modified by carboxyl fusion of degron CL1 (the fusion GFP is known as GFPdgn) have been previously described [21].

The GFPdgn tg mice of approximately 3 months of age were subject to intraperitoneal injections of bortezomib (BZM, 1 mg/kg, i.p.), bafilomycin A1 (BFA, 2.5 mg/kg/12 hrs), or vehicle control (60% DMSO in saline). The mice tolerated the treatment well and no gross abnormality was observed. At 3 hours after BZM injection and at 3 and 24 hours after the first BFA or DMSO injection, the mice were sacrificed through deep anesthesia via isoflurane inhalation and subsequent heart removal. Ventricular and renal tissues were then sampled for total protein and RNA extractions or fixed with 3.8% paraformaldehyde.

### 2. Neonatal rat ventricular cardiomyocyte (NRVM) culture and adenoviral gene delivery

Neonatal rats at postnatal day 0 to day 2 were sacrificed by decapitation and subsequent heart removal. Ventricular myocytes were then isolated using the Cellutron Neomyocytes isolation system (Cellutron Life Technology, Baltimore, MD) following the manufacturer's instructions [22]. Cells collected were suspended in Dulbecco's modified Eagle's medium (DMEM) supplemented with 10% fetal bovine serum (FBS), 100 µM BrdU, and 100 U/ml penicillin/streptomycin (Invitrogen, Carlsbad, CA). To selectively enrich cardiomyocytes, the cells were preplated in 100 mm non-coated dishes for 1 hour. The resulting suspended cells were counted with a hemocytometer and then plated evenly on 1% gelatin-coated plates in appropriate cell densities. The plated cells were then cultured in a 5% CO2 incubator at 37°C for at least 24 hours before the medium was changed to meet the needs of the follow-up experiments.

Creation and validation of recombinant adenoviruses (Ad-) harboring the expression cassette for a red fluorescence protein (RFP) or a GFP with carboxyl fusion of the degron CL1 (GFPu) were previously reported [23]. Infection of cultured NRVMs with any of these recombinant adenoviruses was generally started at 48–72 hours after myocytes were plated. Adenoviral infection of NRVMs was performed in serum-free DMEM in a 5% CO2 incubator at 37°C for 3 hours. Three hours after infection, the cultured media containing the adenovirus were replaced with fresh media containing 2% FBS and the cells were incubated for at least 48 hours to allow the transgene expression.

### 3. Western blot analysis

This was performed as previously described [24]. Crude proteins were extracted from mouse myocardial or renal tissues or cultured NRVMs. The protein concentration was determined using bicinchoninic acid (BCA) reagents (Pierce biotechnology, Rockford, IL). Equal amounts of samples were subjected to sodium dodecyl sulfate -polyacrylamide gel electrophoresis (SDS-PAGE), transferred to PVDF membrane using a Trans-blot apparatus (Bio-Rad, Hercules, CA). The membranes were blocked with 5% non-fat-dry milk in PBS containing 0.1% Tween-20 (PBS-T) for 1 hour at room temperature and then probed with appropriate primary antibodies overnight at 4°C. Primary antibodies against: GAPDH (G8795, Sigma, St Louis, MO), α-actinin (A5044, Sigma), β-tubulin (sc-55529, Santa Cruz), GFP (sc-9996, Santa Cruz Biotechnology), RFP (customized), LC3 (M115-3, Medical & Biological Laboratories Co., MBL, Nagoyo, Japan), p62 (American Research Products, Belmont, MA), and murine proteasome subunit β5 (customized), α3, α4, Rpt6, and β2 (BIOMOL, Plymouth Meeting, PA) were used. The corresponding horseradish peroxidase-conjugated goat anti-mouse, goat anti-rabbit, or goat anti-guinea pig Ig secondary antibodies (Santa Cruz) were respectively used. The bound secondary antibodies were detected using either enhanced chemiluminescence (ECL-Plus) detection reagents (GE Healthcare, Piscataway, NJ) or, for weak signals, ECL Advance Western Blotting Kit (GE Healthcare) and visualized with a VersaDoc3000 imaging system (Bio-Rad). The signal was quantified with the Quantity One software (Bio-Rad).

### 4. Proteasome peptidase activity assays

These assays were done as previously described [25]. Myocardial samples were homogenized at 4°C in 10 volumes HEPES buffer (50 mM, pH 7.5) containing: KCl 20 mM, MgCl2 5 mM, DTT 1 mM. Cell debris was removed by centrifugation for 15 minutes at 12,000×g, and the supernatants were immediately used for protein concentration assay and then determination of peptidase activities. The following synthetic fluorogenic substrates: Suc-LLVY-AMC (18 µM), Z-LLE-AMC (45 µM) (CALBIOCHEM, San Diego, CA), and Ac-RLR-AMC (40 µM, BIOMOL Plymouth Meeting, PA) were used respectively for measuring chymotrypsin-like, caspase-like, and trypsin-like peptidase activities in the absence or presence of a proteasome inhibitor, MG-132 (20 µM, A.G. Scientific, Inc. San Diego, CA) for chymotrypsin-like and caspase-like activities, or epoxomycin (5 µM, CALBIOCHEM, San Diego, CA) for trypsin-like activity. Measurements of each specimen are performed in triplicates. A 5 µg of crude protein extract is added to 200 µl of the HEPES buffer containing the fluorogenic substrate to each well in 96-well plates, and incubated at 37°C. The fluorescence intensity was measured at 60 min of incubation using a Perkin Elmer 2030 Multilabel Microplate Reader with the excitation wave length of 380 nm and emission wave length at 460 nm. The portion of peptidase activity inhibited by the proteasome inhibitor is attributed to the proteasome.

### 5. RNA extraction and reverse transcriptase PCR analysis

Total RNA was extracted from ventricular myocardium using the Tri-Reagent (Molecular Research Center, Inc., Cincinnati, OH, USA) as previously described [26]. The concentration of RNA was determined using Agilent RNA 6000 Nano assay (Agilent technologies, Inc. Germany) following the manufacturer's protocol. For reverse transcription reaction, 1 µg of RNA was used as a template to generate cDNA using the SuperScript III First-Strand Synthesis kit (Invitrogen) and was carried out according to the manufacturer's instructions. For GFPdgn and GAPDH duplex PCR, 2 µl of solution resulting from the reverse transcription reaction and specific primers towards the gene of interest were used. The transcript levels of GPFdgn were quantified by PCR at the minimum number of cycles (20 cycles) that can detect the PCR products within the linear amplification range. The sequences of the specific primers were as follows, GFPdgn: forward 5′-TCT ATA TCA TGG CCG ACA AGC AGA-3′ and reverse 5′-ACT GGG TGC TCA GGT AGT GGT TGT-3′; GAPDH: forward 5′-GCC GTA TTG GGC GCC TGG TCA-3′ and reverse 5′-AAC ATA CTC AGC ACC GGC CTT ACCC-3′. Relative mRNA levels were normalized with GAPDH mRNA levels.

### 6. Direct fluorescence and immunofluorescence confocal microscopy

These were performed as previously described [27], [28]. GFPdgn mouse myocardial tissues were fixed with 3.8% paraformaldehyde (Electron Microscopy Science, Hatfield, PA) by immersion fixation, saturated with 40% sucrose solution, embedded in Tissue-Tek O.C.T. (Sakura Finetek. USA, Inc, Torrance, CA), and frozen and stored in a −80°C freezer. The tissue blocks then underwent cryo-sectioning at 7-micron thickness. The cryosections were permeabilized with 1% of Triton-X100 in PBS for 1 hour and quenched with 0.1 M glycine in PBS for 1 hour. The GFP direct fluorescence in the processed sections visualized using a fluorescence confocal microscope (Olympus Fluoview 500) and the images were captured and digitalized using the associated software.

NRVMs grown on the cover glass were fixed with 3.8% paraformaldehyde for 10 minutes, permeabilized with 1% of Triton-X100 in PBS for 1 hour, quenched with 0.1 M glycine in PBS for 1 hour, and blocked with 0.5% BSA for 1 hour. The specimens were then incubated with primary antibodies overnight at 4°C. The antibody against p62 (American Research products; guinea pig, 1∶100) was used along with Alexa-Fluor 568 donkey anti-guinea pig Ig (Molecular Probes) to immunofluorescence label p62 in the cell. The GFPu direct fluorescence and p62 indirect fluorescence were captured using the fluorescence confocal microscope.

### 7. Cycloheximide (CHX) chase assay

This was performed as previously described [23]. The protein degradation rate of GFPu was tested in the cultured NRVMs after 12 hours of treatment with 3-MA or BFA. Cells were incubated in serum-free DMEM containing 10 µM CHX (Sigma-Aldrich) to block further protein synthesis. In both experiments, cells were collected at immediately before (0 min) or 5, 10, 15, 30, 60, and 120 min after CHX administration; and whole-cell lysates were analyzed for GFPu and RFP by western blot analyses. Band densities were normalized to the one at 0 time point which was set at 1 arbitrary unit (AU).

### 8. Small inference RNA (siRNA) mediated gene knockdown

The siRNA's specific for rat p62 (sip62: 5′- CATGTCCTATGTGAAAGATGA-3′), Atg7 (siAtg7: Cat. # SI01729119), Rab7 (siRab7: Cat. # SI03021725) and the siRNA targeting luciferase serving as a control siRNA (siLuc: 5′-AACGTACGCGGAATACTTCGA-3′) were all purchased from Qiagen (Valencia, CA). LipofectamineTM 2000 transfection reagent (Invitrogen) was used for siRNA transfection following the manufacturer's protocol. Transfection of cultured NRVMs with siRNA was generally started at 48–72 hours after NRVMs were plated. In p62 knock down experiments, the same amounts of luciferase siRNA or p62 siRNA were applied to the control and experimental groups, respectively. Six hours after the transfection, the siRNA-containing medium was replaced with the fresh medium containing 2% FBS. To effectively knock down p62 protein levels, 2 consecutive rounds of transfection of p62 siRNA at a dose of 160 pmol for 2×10^6^ cells with an interval of 72 hours were performed [29]. Three days after the second p62 siRNA transfection, the follow-up assessments were performed. For the Atg7 and Rab7 knockdown experiments, the Atg7 or Rab7 siRNA were introduced along the second round of sip62 or siLuc transfection.

### 9. Statistical analysis

All quantitative data were presented as mean ± S.D. Differences between groups were evaluated for significance using Student's *t*-test for unpaired two group comparison or one-way or two-way ANOVA followed by the Scheffé's test when appropriate. The *p*-value<0.05 is considered statistically significant.

## Results

### 1. Inhibition of the ALP accumulates both autophagic and proteasomal substrates in mice

**Figure 1:**
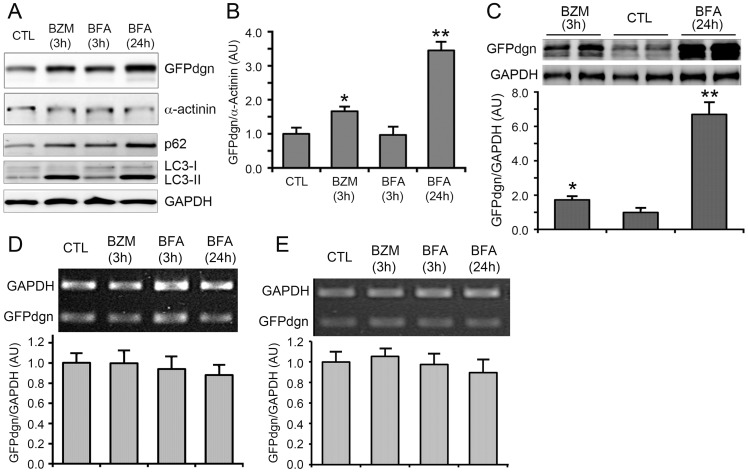
Lysosomal inhibition accumulates both autophagic and proteasomal substrates in mice: hearts and kidneys. Male GFPdgn transgenic mice at 10 weeks of age were treated with bortezomib (BZM, 1 mg/kg, i.p.), bafilomycin A1 (BFA, 2.5 mg/kg/12 hrs, i.p.), or vehicle control (CTL). The mice were sacrificed at either 3 or 24 hours after the first injection and myocardial and kidney tissues were collected for total protein and RNA extraction and subsequent analysis. **A** and **B**, Representative images (A) and pooled of densitometry data (B) of western blot analyses for myocardial GFPdgn and other indicated proteins. **C**, Representative images (upper) and pooled densitometry data (lower) of western blot analyses for renal GFPdgn protein levels. **D** and **E**, Representative images (upper) and pooled densitometry data of reverse transcription (RT) PCR analyses of GFPdgn mRNA levels in heart and kidney tissues. Total RNA was used for the RT to synthesize the first strand cDNA which was subsequently used for GFPdgn and GAPDH duplex RT-PCR. GAPDH was probed as loading control. N = 4 mice/group. *p<0.05, **p<0.01 vs. CTL.

**Figure 2:**
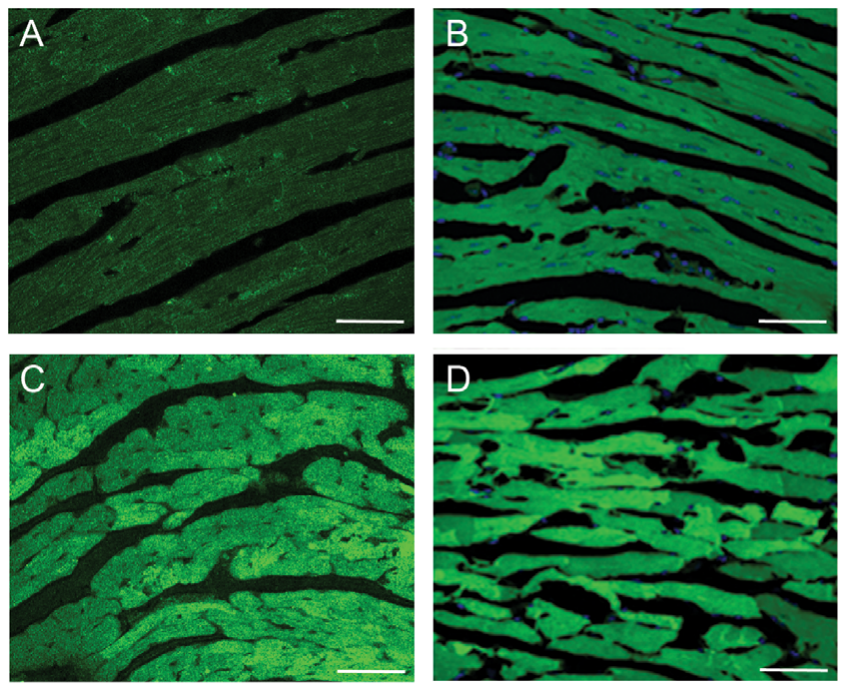
Representative confocal micrographs of mouse myocardial direct green fluorescence. Adult GFPdgn non-transgenic (**A**) and transgenic (**B** ∼ **D**) mice at 10 weeks of age were treated with bortezomib (1 mg/kg, i.p.; **C**), bafilomycin A1 (BFA, 2.5 mg/kg/12 hrs, i.p.; **D**), or vehicle control (DMSO, **A** and **B**). At 24 hours after the first BFA injection, ventricular myocardial tissues were collected and immediately immersion-fixed with 3.8% paraformaldehyde and processed for cryo-sectioning. The cryosections were mounted and imaged for direct fluorescence via confocal microscopy. Scale bar = 100 µm.

It has been previously reported that long-term inhibition of autophagy impairs the degradation of proteasome substrates in cultured cells [19]. To test whether this is the case in intact animals, we treated GFPdgn transgenic mice with bafilomycin A1 (BFA), an inhibitor of the vacuolar proton ATPase that is known to inhibit lysosomal function and the fusion between autophagosomes and lysosomes [30]. Consistent with that GFPdgn is a surrogate UPS substrate [21], [31], myocardial and renal GFPdgn protein levels were significantly increased at 3 hours after administration of a *bona fide* proteasome inhibitor bortezomib (BZM, 1 mg/kg, i.p.); by contrast, myocardial GFPdgn protein levels remained unchanged at 3 hours after the administration of BFA. The effective inhibition of lysosomes by BFA is illustrated by the increases in LC3-II and p62 in the heart of BFA-treated mice. Importantly, myocardial and renal GFPdgn protein levels were significantly increased at 24 hours after the first injection of BFA (2.5 mg/kg/12 h, i.p., **Figure 1A** **∼1C**). The GFPdgn protein increases were not accompanied by discernible alteration in the level of steady state GFPdgn mRNA in either cardiac or renal tissues (**Figure 1D, 1E**), indicating that the increased GFPdgn protein levels occur at a posttranscriptional step. Confocal microscopy showed that the myocardial increases in GFPdgn proteins resided primarily in the cardiomyocytes (**Figure 2**). These data indicate that long-term lysosomal inhibition not only accumulates autophagosomes but also impairs the degradation of a surrogate UPS substrate.

### 2. Myocardial proteasome abundance and intrinsic activities were not altered by lysosomal inhibition

**Figure 3:**
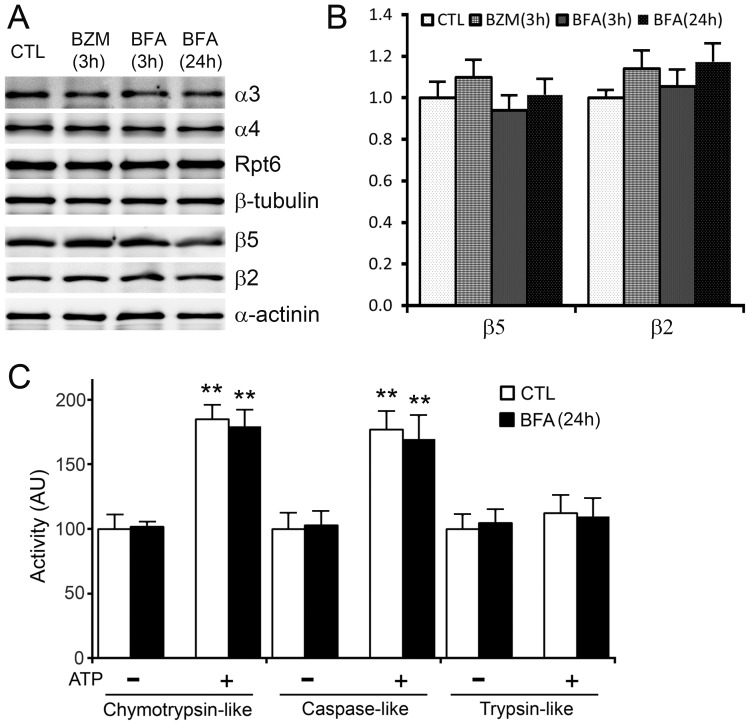
Lysosomal inhibition does not alter proteasome abundance and proteasome peptidase activities in mouse hearts. Mice were treated as described in Figure 1. **A**, Representative images of western blot analyses for the representative subunits of the 19S proteasome (Rpt6) and the 20S proteasome (α3, α4, β2, and β5) in the total protein extracts from ventricular myocardium. **B**, Pooled densitometry data of β2 and β5 subunits as analyzed in A. **C**, Proteasome peptidase activity assays. Synthetic fluorogenic substrates specific for chyomtrypin-like, caspase-like, and trypin-like peptidases were used to measure the proteasomal peptidase activities in the crude protein extracts from ventricular myocardium collected from mice treated with BFA or vehicle control (CTL) for 24 hours. The assay was performed in the absence (-) or presence (+) of ATP to detect the activity of the 20S and the 26S proteasome, respectively. Proteasome inhibitor-suppressible activities were attributed to the proteasome. N = 4 mice/group. **p<0.01 vs. the respective group without ATP.

**G**FPdgn is a surrogate substrate of the UPS and it is constitutively ubiquitinated and degraded by the proteasome. The cardiac accumulation of GFPdgn by long-term lysosomal inhibition prompted us to examine the proteasome abundance and intrinsic peptidase activities in the mouse hearts. Representative subunits of the 19S proteasome (Rpt6) or the 20S proteasomes (α3, α4, β2, and β5) in ventricular myocardium were measured using western blot analyses. The protein abundance of the proteasome subunits was found comparable among the CTL, BZM-3h, BFA-3h, and BFA-24h groups (**Figure 3A and 3B**). More importantly, myocardial chymotrypsin-, caspase-, and trypsin-like peptidase activities attributable to the 20S or the 26S proteasomes were not found to differ significantly between the BFA-treated and the CTL groups (**Figure 3C**). These results indicate that accumulation of GFPdgn by BFA is not due to direct inhibition of proteasome intrinsic proteolytic activities.

### 3. Dynamic effects of ALP inhibition on the protein levels of p62and a surrogate UPS substrate in cultured cardiomyocytes

**Figure 4:**
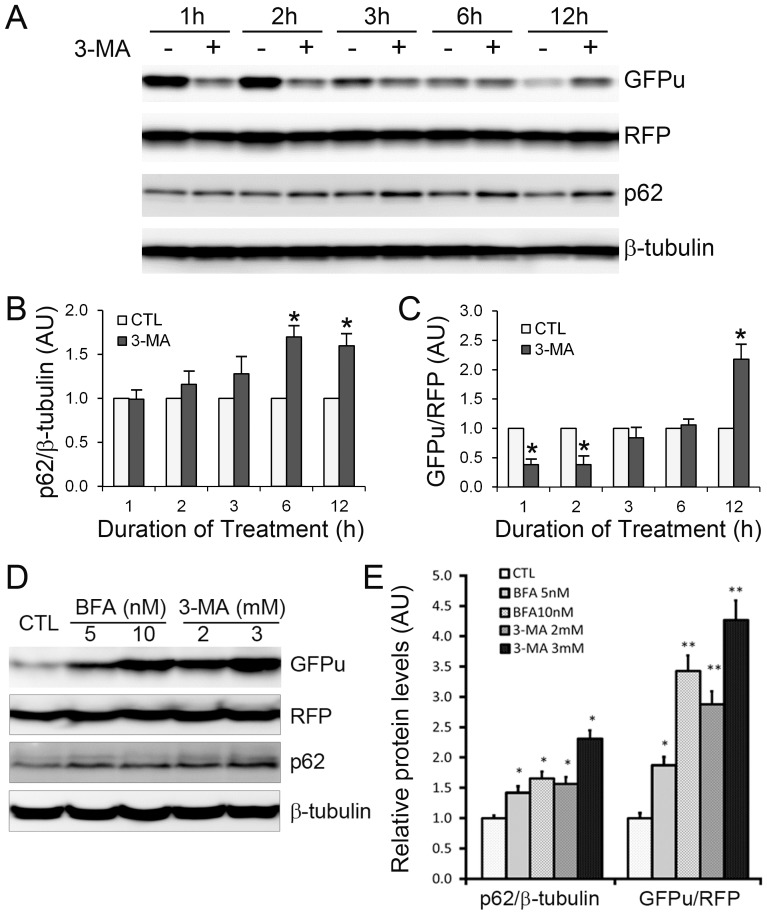
Dynamic effects of ALP inhibition via 3-MA or BFA on p62 protein levels and a surrogate UPS substrate in cultured neonatal rat ventricular myocytes (NRVMs). Cultured NRVMs were infected with adenoviruses to express GFPu and RFP (Ad-GFPu, Ad-RFP) 48 hr before 3-methyladenine (3-MA) or bafilomycin A1 (BFA) treatment. **A ∼ C**, The time course of protein level changes in the GFPu/RFP ratio and the p62/β-tubulin ratio in NRVMs treated with 3-MA (3 mM) or vehicle control. Representative images of western blots (**A**) and quantitative data from 3 repeats (**B, C**) are shown. Paired *t*-test, *p<0.01. **D** and **E**, 3-MA and BFA concentration-dependently increases GFPu/RFP ratio at 24 hours. At 24 hr after the drug treatment, the cells were harvested for total protein extraction and western blot analyses of the indicated proteins. Representative images (**D**) and pooled densitometry data (**E**) are shown. Two-way ANOVA followed by the Scheffé's test; *p<0.05, **p<0.01 vs. CTL; n = 3 repeats/group.

To test whether the effect of ALP inhibition on the degradation of a UPS substrate in the heart is cardiomyocyte-autonomous and is not a secondary response to altered heart function at the organ level or to a systemic neurohumoral factor, we employed a NRVM culture system to investigate the effect of pharmacological or genetic inhibition of the ALP on the UPS. Pharmacologically, 3-methyladenine (3-MA) was used to inhibit the initiation step of macroautophagy or autophagosome formation and bafilomycin A1 (BFA), which inhibits autophagosome-lysosome fusion and lysosomal function, was utilized to inhibit autophagosome removal. UPS performance was evaluated using the GFPu/RFP ratio. Similar to GFPdgn, GFPu is a proven efficient substrate of the UPS;[31], [32] however, RFP is not [33]. To evaluate UPS performance, GFPu and RFP were co-introduced into cultured NRVMs via infection of a mixture of Ad-GFPu and Ad-RFP. The regulatory elements in the GFPu and RFP transgenic constructs are exactly the same so that any changes in transcription and translation will exert the same impact on both GFPu and RFP [33]; hence, the GFPu to RFP steady state protein level ratio (GFPu/RFP) can more accurately reflect inversely UPS performance. The higher the ratio the lower the UPS function. It is well documented that p62 is degraded by macroautophagy.[34] As expected, autophagic inhibition by 3-MA gradually accumulated p62, with p62 protein levels peaking at 6 h of 3-MA treatment and stayed increased at 12 h (**Figure 4A, B**). Interestingly, the GFPu/RFP ratio showed highly dynamic changes during 3-MA treatment. During the first hour of 3-MA treatment, GFPu/RFP ratio dropped remarkably by approximately 60% relative to that of the control group, it remained low at 2 h of treatment but the ratio climbed back to a level comparable to that of the control group at 3 h and 6 h and became significantly higher than the control at 12 h (*p*<0.01, **Figure 4A, C**). Similar results were also obtained from lysosomal inhibition by BFA (*data not shown*). Moreover, both 3-MA and BFA significantly increased the GFPu/RFP ratio at 24 h of treatment (**Figure 4D, 4E**). Notably, similarly to pharmacological inhibition, autophagic inhibition induced by genetic means (i.e., Atg7 or Rab7 knockdown) also increased the GFPu/RFP ratio in cultured NRVMs (*see next section*).

**Figure 5:**
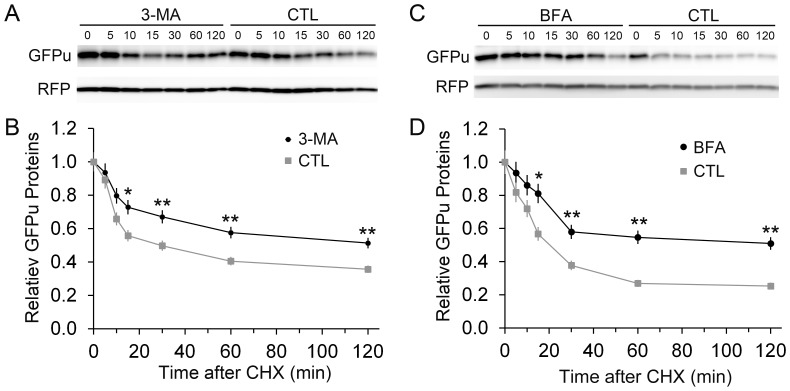
Inhibition of the ALP stabilizes a UPS substrate. NRVMs were cultured and infected with Ad-GFPu/Ad-RFP viral mixture as described in **Figure 4**. Forty-eight hours later, the cells were treated with 3-MA (2 mM, **A** and **B**) or BFA (10 nM, **C** and **D**), or respective vehicle control (CTL) for 12 hrs; then, the treatment of cycloheximide (CHX, 50 µM) was initiated. Immediately before (0 min) or 5, 10, 15, 30, 60, and 120 min after CHX administration, the cells were harvested for western blot analyses for GFPu and RFP. Representative western blot analysis images (**A**, **C**) and densitometry data pooled from 3 biological repeats (**B**, **D**) are shown. *p<0.05, **p<0.01 vs. CTL.

To further verify that GFPu is stabilized by a long-term treatment of 3-MA or BFA, we performed the cycloheximide (CHX) chase assay in the cultured NRVMs. This assay allows monitoring the degradation rate of GFPu when protein synthesis is blocked by CHX. CHX administration was initiated at 12 h after ALP inhibition was started. The protein levels of GFPu and RFP in the total cell lysates immediately before (0 min) or at 5, 10, 15, 30, 60, and 120 min after CHX administration were measured via western blot analyses. These assays showed that after 12 h of treatment of either 3-MA or BFA, the degradation of GFPu was significantly slowed down whereas the degradation of RFP was not discernibly affected (**Figure 5**).

Taken together, these experiments demonstrate that long-term (12 h or longer) but not short term (<6 h) inhibition of the ALP impairs the degradation of proteasome substrates in cardiomyocytes *in vivo* and *in vitro*.

### 4. p62 mediates the ALP inhibition-induced accumulation of proteasome substrates

**Figure 6:**
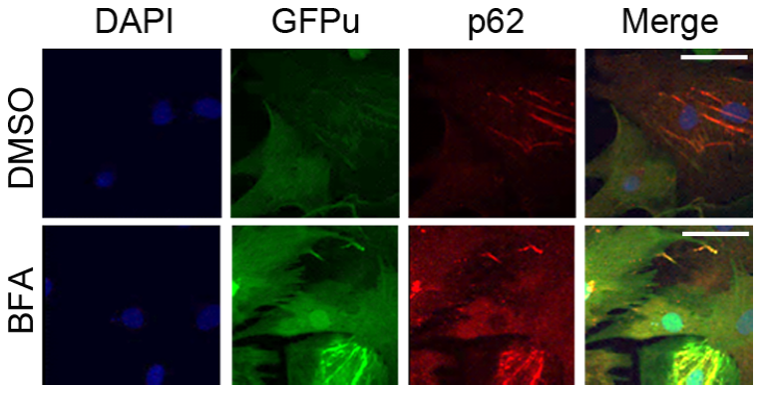
Representative confocal fluorescence micrographs of NRVMs grown on cover glasses. Ad-GFPu infection and BFA (10 nM) treatment are as described in **Figure 4**. At 24 hr after BFA treatment, the cells were fixed with 4% paraformaldehyde and subject to immunofluorescence staining for p62 (red). GFPu direct fluorescence (green) and p62 indirect immunofluorescence were visualized and captured using a confocal microscope. In the BFA treated cells, both p62 and GFPu are increased and the increased GFPu is co-localized with p62. Scale bar = 50 µm.

**Figure 7:**
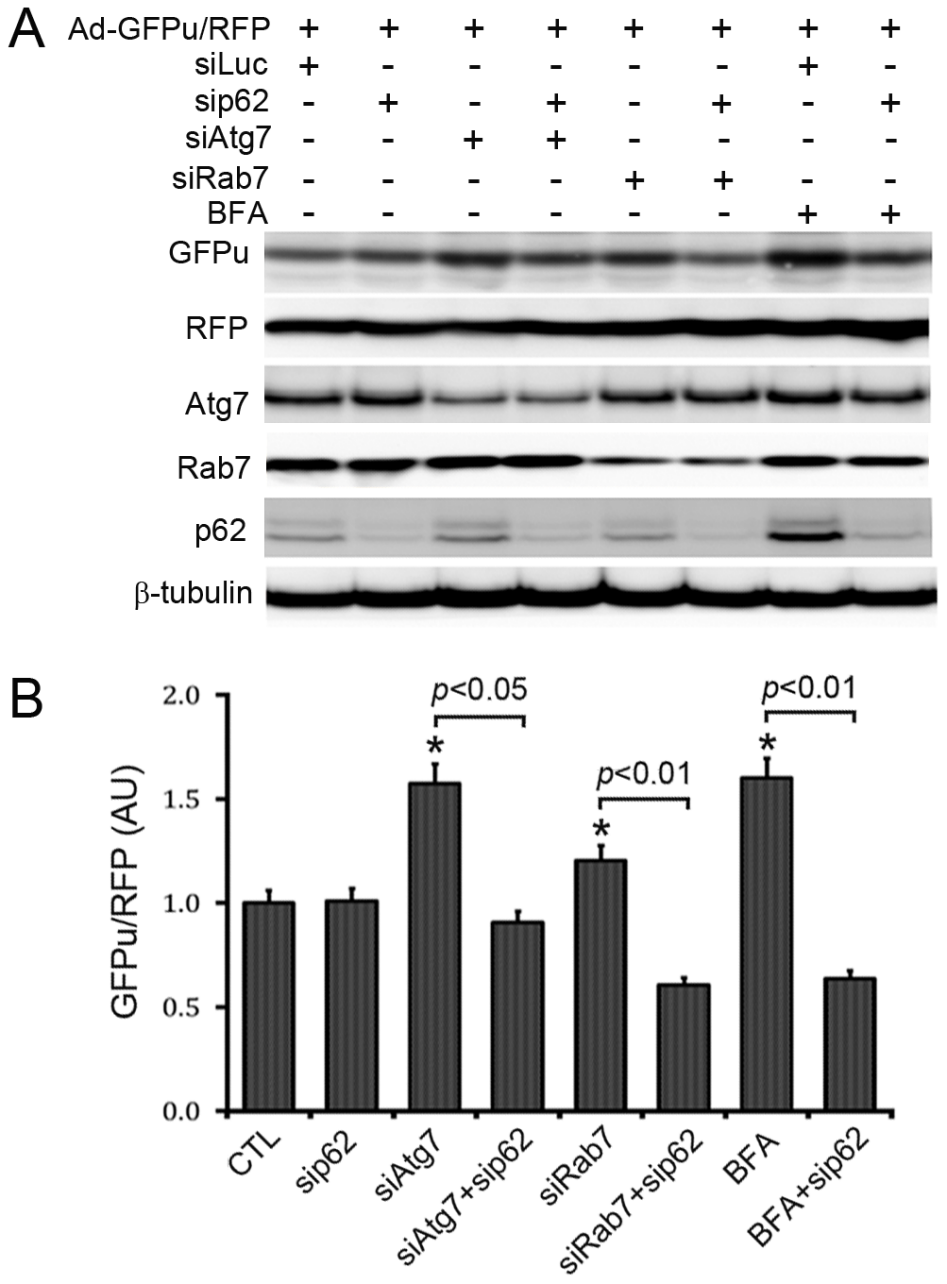
Autophagic inhibition accumulates GFPu in a p62 dependent manner. Cultured NRVMs were infected with adenoviruses to express GFPu and RFP (Ad-GFPu/RFP) 24 hours before transfection with siRNA for Atg7 (siAtg7), Rab7 (siRab7) and/or for p62 (sip62). A luciferase-specific siRNA (siLuc) was used as control for siRNA infection and off-target effects. The cells were harvested at 48 h later for extracting total proteins. **A**, Shown are representative images out of 3 repeats. **B**, pooled densitometry data. *p<0.05 vs. CTL.

Given that the accumulation of p62, a protein capable of binding ubiquitinated proteins, occurs before GFPu stabilization becomes discernible during ALP inhibition (**Figure 4**), we determined the role of p62 in the impairment of UPS performance by long-term ALP inhibition. Confocal microscopy of immunolabeled p62 revealed that the increased GFPu was primarily co-localized with increased p62 in the BFA-treated NRVMs in culture (**Figure 6**). Importantly, GFPu accumulation induced by long-term treatment of BFA was completely blocked by siRNA-mediated specific knockdown of p62 (**Figure 7**).

The product of the autophagy-related gene 7 (Atg7) plays an important role in the formation of autophagosomes and the ablation of Atg7 impairs macroautophagy [35], [36]. Small G-protein Rab7 is critical to the fusion of autophagosomes with lysosomes and perhaps lysosomal genesis [27], [37]–[39]. We have recently shown that Rab7 knockdown decreases autophagic flux via impairment of autophagosome maturation in NRVMs [27]. Hence, we used siRNA-mediated knockdown of either Atg7 or Rab7 as genetic methods to inhibit the ALP. Transfection of the siRNA specific for Atg7 or Rab7 led to significant decreases in the protein levels of Atg7 or Rab7, respectively, compared with control siRNA (luciferase-specific RNA, siLuc) transfection. Importantly, significant increases in the GFPu/RFP ratio were detected at 48 hours after transfection of Atg7 siRNA or Rab7 siRNA whereas the increases were prevented by p62 knockdown (**Figure 7**). These data indicate that p62 mediates the impairment of proteasomal substrate degradation during long-term ALP inhibition.

## Discussion

The effect of ALP inhibition on UPS performance in intact animals has rarely been investigated. Using cultured mammalian non-cardiomyocyte cells, two studies reported so far have yielded opposing results concerning the impact of ALP inhibition on proteasomal function [19], [20]. Little is known about ALP inhibition on UPS-mediated protein degradation in cardiomyocytes. Here we report for the first time that long-term (24 h) but not short-term (3 h) lysosomal inhibition compromises UPS performance in cardiac and renal tissues of intact mice, the two tissues that were examined. We further demonstrate that 12 h or longer ALP inhibition impairs UPS performance and this impairment is p62-mediated in cardiomyocytes. Our findings yield new insight into the interplay between ALP and UPS mediated protein degradation in cardiomyocytes. Giving that inadequacy in both the UPS and the ALP is implicated in at least a subset of human congestive heart failure [3], this study provides new critical information for a better understanding of the molecular basis by which a subset of heart disease progresses to congestive heart failure.

UPS-mediated protein degradation is an ATP-dependent sophisticate process that requires the collaboration of a large number of proteins involved in ubiquitination of the substrate protein, delivery of the ubiquitinated proteins to the proteasome, and proteasomal degradation [40]. Hence, monitoring the steady state level of a UPS substrate protein is the best way to measure UPS performance in a cell or tissue. The endogenous native substrates of the UPS all have their own function in the cell which is highly regulated at transcription, translation, and post-translational levels; hence, their protein levels are often of little value to reflect UPS performance. This is why easily detectable surrogate substrates (e.g., fluorescence proteins engineered for efficient UPS degradation) have been engineered and expressed as constitutive transgenes in cultured cells or animals [21], [32], [41]. The present study took advantage of such a transgenic mouse model (i.e., GFPdgn mice) to reveal that the long term (24 h), but not short term (3 h) of inhibition of the ALP via BFA accumulated GFPdgn proteins but not mRNA in myocardial and renal tissues, the two organs examined (**Figure 1**), providing compelling evidence that ALP inhibition leads to a delayed UPS functional impairment. In the heart, the accumulation of GFPdgn in cardiomyocytes by BFA is further confirmed by myocardial confocal microscopy (**Figure 2**). This decrease in UPS performance was associated with increased p62 protein levels but not altered proteasome abundance or peptidase activity (**Figure 3**). In agreement with our study, Korolchuk *et al*. reported similar findings in cultured Hella cells and mouse embryonic fibroblasts using a different surrogate proteasome substrate [19]. Cathepsin D is an important lysosomal protease [42]. To date the only study on the *in vivo* impact of lysosomal malfunction on proteasomal function was reported by Qiao *et al* [43], which shows that germ-line knockout of the cathepsin D gene impairs macroautophagy and lead to a delayed decrease in proteasome peptidase activities in mouse brains. The proteasome functional deficit induced by cathepsin D deficiency was not discernible until postnatal day 25 and is not accompanied by altered protein expression of multiple proteasome subunits that were examined [43]. Taken together, the impairment of UPS performance by ALP inhibition is caused by factor(s) outside of the proteasome.

At the individual cardiomyocyte level, the impact of ALP inhibition on the global performance of the UPS has not been tested although proteasome chymotrypsin-like activities in NRVMs treated with 3-MA for 7 days were shown to increase significantly [44]. Using NRVM cultures, we observed in the present study that treatment with either 3-MA or BFA significantly increased GFPu/RFP ratio at 12 h or longer (**Figure 4**); moreover, Atg7 knockdown and Rab7 knockdown, both of which inhibited the ALP, also increased the GFPu/RFP ratio (**Figure 7**). Our CHX chase assays further confirmed that ALKP inhibition via 3-MA or BFA indeed stabilizes GFPu protein, a *bona fide* UPS substrate (**Figure 5**). These results indicate compellingly that long-term ALP inhibition impairs UPS performance in cardiomyocytes. This is not only consistent with our findings from GFPdgn tg mice but also suggest that cardiac UPS impairment resulted from ALP inhibition via BFA treatment in mice is cardiomyocyte-autonomous, not a secondary response to systemic lysosomal malfunction. Our findings from NRVM cultures appear to differ from what reported recently for colon cancer cell lines by Wang *et al* [20]. They showed that ALP inhibition by either chloroquine treatment or knocking down the Atg5 or Atg7 gene induced significant increases in proteasome subunit expression and proteasomal peptidase activities in cultured SW1116 and HCT116 cells and the induction of proteasomal activity was corroborated by enhanced degradation of ubiquitin-independent substrates. Unfortunately, the impact of ALP inhibition on proteasomal removal of ubiquitin-dependent substrates was not tested in the colon cancer cell lines [20]. The proteasomal degradation of our reporter protein GFPu is ubiquitin-dependent. However, our *in vivo* ALP inhibition experiments did not observed changes in proteasome expression and activities, which is in sharp contrast to the report with colon cancer cell lines. It is unclear what causes this discrepancy but difference in the degree of differentiation between heart muscle cells and cancer cells may be a potential cause. Notably, in agreement with our findings, Komatsu *et al*. reported that neuron-specific ALP inhibition by Atg7 knockout accumulates ubiquitinated proteins without discernible changes in proteasome activities in the mouse brain [45].

Bearing both a ubiquitin binding domain (UBA) and an LC3 interacting motif (LIR), p62 is purported to target aberrant protein aggregates and defective organelles (e.g., mitochondria) which carry poly-ubiquitin moieties suitable for UBA to bind, to autophagosomes for ALP-mediated removal [46], [47]. Increased ubiquitinated proteins resulting from proteasome malfunction are expected to stabilize p62. Plus, the synthesis of p62 in a proteasome inhibited cell is likely increased as the transcription of p62 has been shown to significantly increase in mouse hearts with PFI [29]. As expected, we observed increased p62 protein levels in mouse hearts treated with a proteasome inhibitor BZM in the present study and, not surprisingly, p62 as an ALP substrate was apparently accumulated by BFA-induced ALP inhibition in mouse hearts (**Figure 1A**). In non-cardiac cells, p62 accumulation was found to be responsible for impaired degradation of UPS substrates [19]. Here we have demonstrated this is also the case in cardiomyocytes. First, our CHX chase assays show the increases in GFPu/RFP by ALP inhibition in cardiomyocytes is indeed caused by a posttranslational stabilization of the GFPu protein (**Figure 5**); second, increased GFPu and p62 co-localize in ALP inhibited cardiomyocytes (**Figure 6**); and lastly ALP inhibition by either pharmacologically (BFA) or genetically (siRNA mediated Atg7 or Rab7 knockdown) could no longer increase the GFPu/RFP ratio in the cardiomyocytes with siRNA-mediated p62 knockdown (**Figure 7**).

Presently, it is unclear how exactly p62 accumulation impairs UPS performance in cardiomyocytes but p62 is known to bind polyubiquitinated proteins via its UBA domain and to self-oligomerize via its PB1 domain [17], [46]. Hence, it is conceivable that p62 accumulation promotes individual ubiquitinated proteins, which would be targeted to the proteasome for degradation, to form oligomers and become inaccessible to the proteasome. Normally, this process may channel ubiquitinated proteins to the ALP for bulky removal [46]; however, upon ALP inhibition or impairment, this prevents ubiquitinated proteins from degradation by the proteasome, and thereby disrupting proteostasis in the cell. Indeed, we recently observed that impaired autophagosome maturation and resultant decreased autophagic flux occur before impaired UPS performance becomes discernible in the COP9 signalosome subunit 8 (CSN8) deficient mouse hearts [27].

Taken together, our data suggest that long-term inhibition of the ALP impairs the degradation of UPS substrates in a p62 dependent manner. The implication of this discovery is potentially broad and significant for both basic and translational research. For example, this may help explain why most lysosomal storage diseases are precipitating in nature; evidence of UPS malfunction and lysosomal deficiency was found to co-exist in failing human hearts [3], [48]; and measures to improve autophagy may indirectly help alleviate UPS insufficiency.
